# Safety, Adherence and Persistence in a Real-World Cohort of German MS Patients Newly Treated With Ocrelizumab: First Insights From the CONFIDENCE Study

**DOI:** 10.3389/fneur.2022.863105

**Published:** 2022-05-09

**Authors:** Martin S. Weber, Mathias Buttmann, Sven G. Meuth, Petra Dirks, Erwan Muros-Le Rouzic, Julius C. Eggebrecht, Stefanie Hieke-Schulz, Jost Leemhuis, Tjalf Ziemssen

**Affiliations:** ^1^Department of Neurology, Institute of Neuropathology, University Medicine Göttingen, Göttingen, Germany; ^2^Fraunhofer Institute for Translational Medicine and Pharmacology ITMP, Göttingen, Germany; ^3^Caritas-Hospital, Bad Mergentheim, Germany; ^4^Clinic of Neurology, Heinrich-Heine University, Düsseldorf, Germany; ^5^F. Hoffmann-La Roche Ltd, Basel, Switzerland; ^6^Roche Pharma AG, Grenzach-Wyhlen, Germany; ^7^Center of Clinical Neuroscience, Neurological Clinic, Carl Gustav Carus University Clinic, University of Technology, Dresden, Germany

**Keywords:** neurodegenerative diseases, multiple sclerosis, non-interventional study (NIS), real-world cohort, safety, drug (or treatment) persistence, humanized monoclonal antibody anti-CD20, ocrelizumab

## Abstract

**Background:**

Real-world relapsing multiple sclerosis (RMS) and primary progressive MS (PPMS) populations may be more diverse than in clinical trials. Here, we present a first analysis of safety, adherence and persistence data from a real-world cohort of patients newly treated with ocrelizumab.

**Methods:**

CONFIDENCE (ML39632, EUPAS22951) is an ongoing multicenter, non-interventional post authorization safety study assessing patients with RMS or PPMS newly treated with ocrelizumab or other disease-modifying therapies for up to 10 years. For this analysis, patients newly treated with ocrelizumab were analyzed in subgroups by MS phenotype and age over a mean ~1 year of exposure totaling 2,329 patient years [PY]).

**Results:**

At data cutoff (14 October 2020), 1,702 patients with RMS and 398 patients with PPMS were treated with ≥1 dose of ocrelizumab. At baseline, the mean ages (SD) of patients with RMS and PPMS were 41.59 (11.24) and 50.95 (9.88) years and the mean EDSS (Expanded Disability Status Scale) was 3.18 (1.87) and 4.41 (1.59), respectively. The most common adverse events (AEs) and serious AEs across both phenotypes were infections and infestations, with infection SAE rates of 2.8 events/100 PY and 1.5 events/100 PY in patients with RMS and PPMS, respectively. Across all phenotypes, ocrelizumab persistence was 92% at 24 months; median time between doses was ~6 months.

**Conclusions:**

The ocrelizumab safety profile observed in the CONFIDENCE real-world MS population was consistent to the one observed in pivotal clinical trials. High treatment persistence and adherence were observed.

**Trial Registration:**

ML39632, EUPAS22951

## Introduction

Multiple sclerosis (MS) is a chronic disease of the central nervous system (CNS) with a complex immunopathogenesis of inflammation and neurodegeneration with two major disease phenotypes: relapsing MS (RMS) and primary progressive MS (PPMS). MS often warrants long-term drug therapy. Thus, the benefits of a disease-modifying therapy (DMT) must outweigh its long-term risks (1).

Ocrelizumab (Ocrevus®) is a monoclonal antibody that specifically binds to CD20, modulating the immunopathogenesis of MS by depleting CD20^+^ B-cells. As the first anti-CD20 monoclonal antibody approved for the treatment of RMS and PPMS, ocrelizumab remains the only approved treatment for PPMS to date (2, 3). Pivotal trial data shows that treatment with ocrelizumab has significant effects on slowing disease progression, annualized relapse rate and magnetic resonance imagery outcomes, with no signal of a higher rate of serious infections, compared with interferon in patients with RMS and placebo in patients with PPMS (4, 5). Ocrelizumab efficacy was sustained in the open-label extension phases of the pivotal trials, where adverse events (AEs) were generally consistent with those from the controlled periods and no new safety signals emerged with prolonged treatment (6, 7).

Integrated safety analysis of the data from 11 clinical trials and open-label extension periods (up to 7 years of continuous ocrelizumab treatment) demonstrated a favorable and manageable safety profile (8). There was no indication of higher rates of malignancy compared with matched reference MS and general populations over 8 years (8).

Real-world populations may be more diverse than those included in randomized clinical trials (RCTs), comprising patients with more prior MS-specific treatments, a longer duration of disease, more physical disability, older age (9) or more comorbidities that may affect safety of treatment (10).

CONFIDENCE (ML39632, EUPAS22951) is a large, ongoing, non-interventional post-authorization safety study (PASS) that assesses the long-term safety and effectiveness of ocrelizumab and other DMTs in a real-world MS population in Germany (11). As the central study of the ocrelizumab post-marketing safety program, safety data from CONFIDENCE is integrated into the two multi-source, real-world studies VERISMO (EUPAS30752) and MANUSCRIPT (EUPAS28619). Here, we present an analysis of baseline characteristics, safety, adherence and persistence from patients newly treated with ocrelizumab in CONFIDENCE over a mean of ~1 year of exposure (max 2.5 years).

## Materials and Methods

### Study Design

CONFIDENCE assesses the long-term safety and effectiveness of patients newly treated with ocrelizumab or other selected DMTs (i.e., alemtuzumab, cladribine, dimethyl fumarate, fingolimod, natalizumab, or teriflunomide). Recruitment was initiated in April 2018 with a target enrollment of 3,000 patients with RMS (relapsing remitting MS or relapsing secondary progressive MS) or PPMS newly treated with ocrelizumab and 767 patients with RMS newly treated with other selected DMTs at ~185 centers in Germany. Full details on the CONFIDENCE study design and inclusion/exclusion have been previously published (11).

The decision to prescribe the treatment must be made prior to and independent of participation in this study; patients are to be treated according to local label. Key inclusion criteria are ≥18 years of age at enrollment and treatment with ocrelizumab or the respective DMT for the first time during the course of MS therapy. Patients participating in an interventional study examining a MS DMT and patients previously treated with rituximab or any other anti-CD20 antibody for MS are excluded.

Overall, patients will be observed with regular ~6-month follow-up visits for up to 10 years regardless of treatment change. CONFIDENCE completion is expected in 2029. Data are collected by site staff and entered into an electronic case report form (eCRF) based on the MS management system 3D (MSDS3D; MedicalSyn, Stuttgart, Germany) (12, 13). Patient demographics and informed consent are collected at screening. Other baseline characteristics such as MS disease and treatment history, general medical history and comorbidities (previous and current diseases and disorders in the patient's medical history), pregnancy status and history, malignancy risk factors, cancer screening and MS disease activity are documented at the baseline visit (first ocrelizumab administration).

Here, we present a first analysis of safety, adherence and persistence of patients treated with ocrelizumab (data cutoff 14 October 2020). Other DMT cohorts were not included in this analysis due to insufficient patient numbers.

### Safety Endpoints

AEs and serious AEs (SAEs) were recorded according to system organ class (SOC) and preferred term (PT) of the Medical Dictionary for Regulatory Activities (MedDRA; version 23.1). As of a protocol amendment in July 2019, infusion-related reactions (IRRs) were only to be recorded if judged as serious or life-threatening. Malignancies were identified using the SOC “Neoplasms benign, malignant and unspecified (incl. cysts and polyps)” and further defined using the standardized MedDRA queries (SMQ) “Malignant tumor (narrow)”. Reasons for discontinuations were reported by the investigator from multiple choices. No specific details were collected for reasons such as “patient wish” or “insufficient efficacy”. Adjunct data from the Roche safety database was used to consummate patient narratives.

### Trial Registration and Ethics Statement

This study was registered on 06 March 2018 in the EU PAS Register (http://www.encepp.eu/encepp/studiesDatabase.jsp) under the EU PAS Register Number EUPAS22951. The independent ethics committee at the Technical University Dresden has given the first professional advice for this observational study (Ethikkommission an der Technischen Universität Dresden, Germany; 12 February 2018 and 10 April 2019; reference EK 62022018). Obtaining further ethics approvals was the individual responsibility of the participating physicians.

### Statistical Analysis

This analysis was based on data prior to the cutoff. Patients who received at least one dose of ocrelizumab were included for analysis of all safety endpoints (safety analysis set). Persistence and adherence endpoints were analyzed in patients in the safety analysis set with at least one documentation after start of the therapy (full analysis set). Persistence was estimated by Kaplan-Meier time-to-treatment discontinuation, in which patients without discontinuation were censored with their last assessment visit date recorded. Adherence was evaluated by median time interval (interquartile range) between dosing. All outcomes were assessed using descriptive statistics. Analysis of patients with PPMS >55 years old at baseline was prespecified in the statistical analysis plan; patients with RMS >55 years old were assessed in a *post-hoc* analysis.

## Results

### Patient Population and Treatment Exposure

As of the data cutoff, 1,702 patients with RMS and 398 patients with PPMS have been treated with ≥1 dose of ocrelizumab and were included in the safety analysis. The mean exposure time (standard deviation, SD) to ocrelizumab was 1.03 (0.70) years for patients with RMS (range 0.0–2.5 years; totaling 1,877 patient-years [PY]) and 1.06 (0.68) years for patients with PPMS (range 0.0–2.5 years; totaling 452 PY).

Mean age (SD) of patients with RMS was 41.59 (11.24) years, 66.9% were females and 82.7% had ≥1 MS-specific prior therapy. The mean (SD) baseline EDSS (Expanded Disability Status Scale) of patients with RMS was 3.18 (1.87) in the total cohort, and 4.54 (1.64) in patients >55 years old. At baseline, 66.0% of ocrelizumab-treated patients with RMS had comorbidities. The most common comorbidities (PT) of patients with RMS were vitamin D deficiency, hypertension and depression (Table 1). In patients with RMS >55 years old, 80.5% had comorbidities, with the most common (PT) being hypertension (Table 1).

**Table 1:** Baseline characteristics (safety set).

**Characteristic**	**Total RMS (*n =* 1,702)**	**RMS >55 years (*n =* 200)**	**Total PPMS (*n =* 398)**	**PPMS >55 years (*n =* 143)**
Age, mean (SD), years	41.59 (11.24)	59.9 (4.12)	50.95 (9.88)	60.90 (4.80)
Sex, n (%)				
Female	1,139 (66.9)	118 (59.0)	208 (52.3)	82 (57.3)
Number of prior MS therapies, n (%)				
Treatment-naïve	294 (17.3)	41 (20.5)	268 (67.3)	102 (71.3)
1	410 (24.1)	45 (22.5)	71 (17.8)	22 (15.4)
2	411 (24.1)	49 (24.5)	32 (8.0)	8 (5.6)
≥3	587 (34.5)	65 (32.5)	27 (6.8)	11 (7.7)
Therapy prior to ocrelizumab, n (%)				
Fingolimod	339 (19.9)	38 (19.0)	11 (2.8)	1 (0.7)
Interferon or GA	274 (16.1)	34 (17)	51 (12.8)	13 (9.1)
Natalizumab	246 (14.5)	11 (5.5)	8 (2.0)	4 (2.8)
Dimethyl fumarate	222 (13.0)	17 (8.5)	19 (4.8)	5 (3.5)
Other/none	621 (36.5)	100 (50.0)	309 (77.6)	120 (83.9)
EDSS, mean (SD)	3.18 (1.87)	4.54 (1.64)	4.41 (1.59)	4.73 (1.48)
Duration to baseline since^*^:				
First symptoms, mean (SD), years	10.79 (8.69)	17.95 (11.71)	8.66 (7.62)	10.63 (9.26)
Diagnosis, mean (SD), years	8.95 (7.81)	14.12 (10.05)	5.60 (6.75)	6.90 (8.39)
Common comorbidities SOC, n (%)				
≥1	1,123 (66.0)	161 (80.5)	296 (74.4)	123 (86.0)
Metabolism and nutrition disorders	430 (25.3)	62 (31.0)	108 (27.1)	41 (28.7)
Nervous system disorders	367 (21.6)	59 (29.5)	90 (22.6)	41 (28.7)
Psychiatric disorders	326 (19.2)	49 (24.5)	71 (17.8)	32 (22.4)
Vascular disorders	235 (13.8)	71 (35.5)	106 (26.6)	56 (39.2)
Endocrine disorders	196 (11.5)	31 (15.5)	46 (11.6)	19 (13.3)
Common comorbidities, PT, n (%)				
Vitamin D deficiency	305 (17.9)	33 (16.5)	57 (14.3)	23 (16.1)
Hypertension	209 (12.3)	59 (29.5)	96 (24.1)	51 (35.7)
Depression	197 (11.6)	30 (15)	40 (10.1)	21 (14.7)

* *Data collected retrospectively*.

*GA, glatiramer acetate; PPMS, primary progressive multiple sclerosis; RMS, relapsing MS; SD, standard deviation; PT, preferred term; SOC, system organ class. Data were analyzed in the safety set, which included all enrolled patients with at least one dose of ocrelizumab*.

*Common comorbidities listed are those in ≥10% of patients with RMS*.

Patients with PPMS were mean (SD) 50.95 (9.88) years old, 52.3% female, and 32.6% had ≥1 MS-specific prior therapy (Table 1). Patients with PPMS had a mean (SD) baseline EDSS of 4.41 (1.59) and 4.73 (1.48) in patients >55 years old. At baseline, 74.4% of patients with PPMS had comorbidities, most commonly (PT) hypertension and vitamin D deficiency. In patients with PPMS > 55 years, 86.0% had comorbidities with the most common (PT) being hypertension (Table 1).

### Adverse Events

In this analysis, 721 (42.4%) ocrelizumab-treated patients with RMS experienced 2,182 AEs [116.2 events/100 PY], most commonly categorized as infections and infestations [32 events/100 PY]. The most common AEs were nasopharyngitis [8.3 events/100 PY], urinary tract infections [6.2 events/100 PY] and infusion-related reactions [5.4 events/100PY] (for a list of all SOCs and the three most common AEs, please see Supplementary Table 1). Overall, 146 (8.6%) patients experienced 250 SAEs [13.3 events/100 PY], the most common being categorized as infections and infestations (Table 2). The most common SAE was urinary tract infection [0.7 events/100 PY]. Of patients with RMS >55 years old (*n* = 200), 86 patients experienced ≥1 AE, [108.7 events/100 PY], most commonly categorized as infections and infestations [29.3 events/100 PY]. The most common AEs were urinary tract infection [8.3 events/100 PY] and infusion-related reactions [5.4 events/100 PY]. Twenty-three (11.5%) RMS patients >55 years experienced 55 total SAEs [22.7 events/100 PY], most commonly categorized as injury, poisoning and procedural complication [4.1 events/100 PY]. The most common SAEs were urinary tract infection, fall and trigeminal neuralgia [all 1.2 events/100 PY].

**Table 2:** Adverse events (AEs), serious AEs, malignancies, infections and serious infections observed in patients treated with ocrelizumab.

	**Total RMS (*n =* 1,702)**				**RMS >55 years (*n =* 200)**				**Total PPMS (*n =* 398)**				**PPMS >55 years (*n =* 143)**			
**Exposure in PY**	**1,877**				**242**				**452**				**162**			
	**Total AEs**		**SAEs**		**Total AEs**		**SAEs**		**Total AEs**		**SAEs**		**Total AEs**		**SAEs**	
	E^**^	R^**^	E^**^	R^**^	E^**^	R^**^	E^**^	R^**^	E^**^	R^**^	E^**^	R^**^	E^**^	R^**^	E^**^	R^**^
Any AE	2,186	116	250	13.3	263	109	55	22.7	380	84	37	8.2	169	104	19	11.7
Fatal events	3	0.2	3	0.2	-	-	-	-	2	0.4	2	0.4	2	1.2	2	1.2
Malignancies^***^	9	0.5	9	0.5	-	-	-	-	3	0.7	3	0.7	1	0.6	1	0.6
Infections																
Nasopharyngitis	155	8.3	-	-	12	5.0	-	-	26	5.8	-	-	11	6.8	-	-
Urinary tract infection	116	6.2	13	0.7	20	8.3	3	1.2	22	4.9	2	0.4	8	4.9	-	-
Upper respiratory	35	1.9	1	0.05	4	1.7			3	0.7	-	-	2	1.2	-	-
tract infection											
Respiratory tract infection	26	1.4	-	-	3	1.2	-	-	2	0.4	-	-	1	0.6	-	-
Bronchitis	23	1.2	1	0.05	4	1.7	-	-	2	0.4	-	-	2	1.2	-	-
Sinusitis	22	1.2	3	0.2	3	1.2	1	0.4	2	0.4	-	-	-	-	-	-
Gastrointestinal infection	20	1.1	-	-	-	-	-	-	4	0.9	-	-	2	1.2	-	-
Oral herpes	15	0.8	-	-	1	0.4	-	-	2	0.4	-	-	-	-	-	-
Herpes zoster	12	0.6	1	0.05	2	0.8	-	-	3	0.7	-	-	2	1.2	-	-
Pneumonia	9	0.5	6	0.3	1	0.4	-	-	2	0.4	2	0.4	-	-	-	-
COVID-19^****^	6	0.3	2	0.1	-	-	-	-	-	-	-	-	-	-	-	-
PML	1	0.05	1	0.05												

* *Total events*.

** *Rate, AEs/100 PY, calculated by dividing total AEs by exposure in 100 PY*.

*** *Malignant tumor (narrow); a full list of the MedDRA SOC “Neoplasms benign, malignant and unspecified (incl cysts and polyps)” is included in Supplementary Table 3*.

**** *Includes COVID-19 and COVID-19 pneumonia*.

*PML, Progressive multifocal leukoencephalopathy. E, Total number of events; PY, patient years; R, rate of events by PY. Infections and serious infections according to MedDRA SOC “Infections and infestations”. A list of all “Infections and infestations” is included in Supplementary Table 2. Data were analyzed in the safety set, which included all enrolled patients with at least one dose of ocrelizumab; AEs were classified according to MedDRA version 23.1. Table data includes all infections ≥0.5 events/100 PY in patients with RMS while COVID-19 and PML were included as infections of interest*.

Overall, 147 (36.9%) ocrelizumab-treated patients with PPMS experienced 380 AEs [84.1 events/100 PY], which were most often categorized as infections and infestations [19.7 events/100 PY] (Table 2). The most common AEs were nasopharyngitis [5.8 events/100 PY] and urinary tract infection [4.9 events/100 PY]. There were 37 SAEs in 26 (6.5%) patients with PPMS [8.2 events/100 PY]. SAEs were most often categorized in the SOC infections and infestations [1.5 events/100 PY]. The most common SAEs were muscle spasticity (3 patients) and fall (3 patients). Of patients with PPMS >55 years old (*n* = 143), 39.9% experienced 169 AEs [104 events/100 PY], most commonly categorized as infections and infestations [21.6 events/100 PY]. The most common AEs were nasopharyngitis [6.8 events/100 PY] and urinary tract infections [4.9 events/100 PY]. Thirteen (9.1%) >55 year-old patients with PPMS experienced 19 SAEs [11.7 events/100 PY]; no additional patterns in reported SAEs were observed.

### Infections and Infestations

Overall, 21.0% of patients with RMS experienced infections [32.2 events/100 PY]. The most common infections were nasopharyngitis [8.3 events/100 PY], urinary tract infections [6.2 events/100 PY] and respiratory tract infections (for a list of all infections, please see Supplementary Table 2). Serious infections were experienced by 2.5% of patients with RMS [2.8 events/100 PY] (Table 2), including 13 events of serious urinary tract infections [0.7 events/100 PY; 12 recovered/recovering and one unknown outcome] and six events of serious pneumonia [0.3 events/100 PY; all recovered/recovering] (Table 2).

A single case of suspected carry-over progressive multifocal leukoencephalopathy (PML), associated with prior natalizumab therapy, was reported in 2018. The case was assessed by an independent panel of PML experts and was classed as suspected rather than confirmed carry-over PML. The patient had magnetic resonance imaging findings suggestive of PML, but the cerebrospinal fluid was negative for JC virus DNA and no clinical symptoms consistent with PML were reported, therefore, the case did not meet the American Association of Neurology criteria for confirmed PML (14). No further cases of PML have been reported in this study. COVID-19 was recorded for 6 patients with RMS [0.3 events/100 PY], 2 of which were considered SAEs [0.1 events/100 PY]. One 44-year-old female was hospitalized due to COVID-19, and one case of serious COVID-19 was of ‘moderate' severity. Both patients recovered. Mini-narratives of SAE infections of interest such as herpes zoster, neuroborreliosis, meningitis, endocarditis, suspected PML and COVID-19 are included in the Supplementary Material.

Five patients with RMS who experienced seven serious infections were >55 years old [2.9 events/100 PY]; these included urinary tract infection (3, all recovered/recovering); urosepsis (2, recovered and unknown outcome); sinusitis (1, recovered); and viral pharyngitis (1, recovered).

Overall, 15.8% of patients with PPMS experienced infections [19.7 events/100 PY], including nasopharyngitis [5.8 events/100 PY] and urinary tract infections [4.9 events/100 PY]. Seven patients with PPMS [1.5 events/100 PY] had serious infections and infestations, (2 pneumonia, recovered and recovered with sequelae; 2 urinary tract infections, recovered and unknown outcome; 1 diverticulitis, recovered; 1 encephalitis, recovered; and 1 urosepsis, recovered). Among patients with PPMS and >55 years old, one case of diverticulitis and one case of encephalitis occurred [1.2 events/100 PY] (for further details see Supplementary Table 4).

### Fatal Events

Three patients with RMS [0.2%; 0.2 events/100 PY; ≤ 55 years old] and 2 patients with PPMS [0.5%; 0.4 events/100 PY; >55 years old] died. Among the patients with RMS, one event was reported as “death” with no specific cause given; one patient—who had a history of tobacco use—died of bronchial carcinoma, and one patient died of myocarditis. In patients with PPMS, one event was reported as “death” (not further specified), and one patient with PPMS completed suicide (for further details see the Supplementary Material).

### Malignancies

Seven patients with RMS experienced malignancies, defined as standardized MedDRA queries (SMQ) “Malignant tumor (narrow)” [0.4%; 0.5 events/100 PY; all ≤ 55 years old]. These included female breast cancer (two cases, 54 and 53 years old at baseline), malignant melanoma (two cases; one including metastases to the mediastinum; 38 and 45 years) and one case each of bronchial carcinoma (54 years), thyroid cancer (52 years) and basal cell carcinoma (42 years). Six of seven patients with a malignancy were female.

Three patients with PPMS experienced a malignancy [0.8%; 0.7 events/100 PY], including malignant melanoma (55 years old at baseline), squamous cell carcinoma of the skin (60 years) and basal cell carcinoma (44 years). Two patients were female, and one was male. Further information on patient history and risk factors can be found in the Supplementary Material.

### Discontinuation, Persistence, and Adherence

Of patients with RMS, 80 (4.7%) discontinued ocrelizumab; the most common reasons were “patient wish” (37), AE (13) and “insufficient efficacy” (12). Fourteen patients >55 years old (7.0%) discontinued, most commonly due to “patient wish” (7), AE (3) and “insufficient efficacy” (3) (Table 3).

**Table 3:** Reasons for discontinuation of ocrelizumab as reported by the investigator.

Reason for discontinuation^*^, n (%)	**Total RMS (*n =* 1,702)**	**RMS >55 years (*n =* 200)**	**Total PPMS (*n =* 398)**	**Total PPMS >55 years (*n =* 143)**
Total discontinuation	80 (4.7)	14 (7.0)	19 (4.8)	10 (7.0)
Reasons for discontinuation				
Patient wish	37 (2.2)	7 (3.5)	9 (2.3)	4 (2.8)
Adverse event	13 (0.8)	3 (1.5)	4 (1.0)	3 (2.1)
Insufficient efficacy^**^	12 (0.7)	3 (1.5)	3 (0.8)	2 (1.4)
Pregnancy wish	6 (0.4)	-	-	-
Pregnancy	4 (0.2)	-	-	-
Other	8 (0.5)	1 (0.5)	3 (0.8)	1 (0.7)

*Data were analyzed in the safety set, which included all enrolled patients with at least one dose of ocrelizumab*.

* *Only one reason was given per patient*.

** *Insufficient efficacy as reported by the investigator, not further specified*.

Of patients with PPMS, 19 (4.8%) discontinued ocrelizumab; the most common reasons were “patient wish” (9), AE (4) and “insufficient efficacy” (3). Ten patients >55 years old (7.0%) discontinued, most commonly due to “patient wish” (4), AE (3) and “insufficient efficacy” (2) (Table 3). Known AEs that led to discontinuation are listed in Supplementary Table 5.

**Figure 1:**
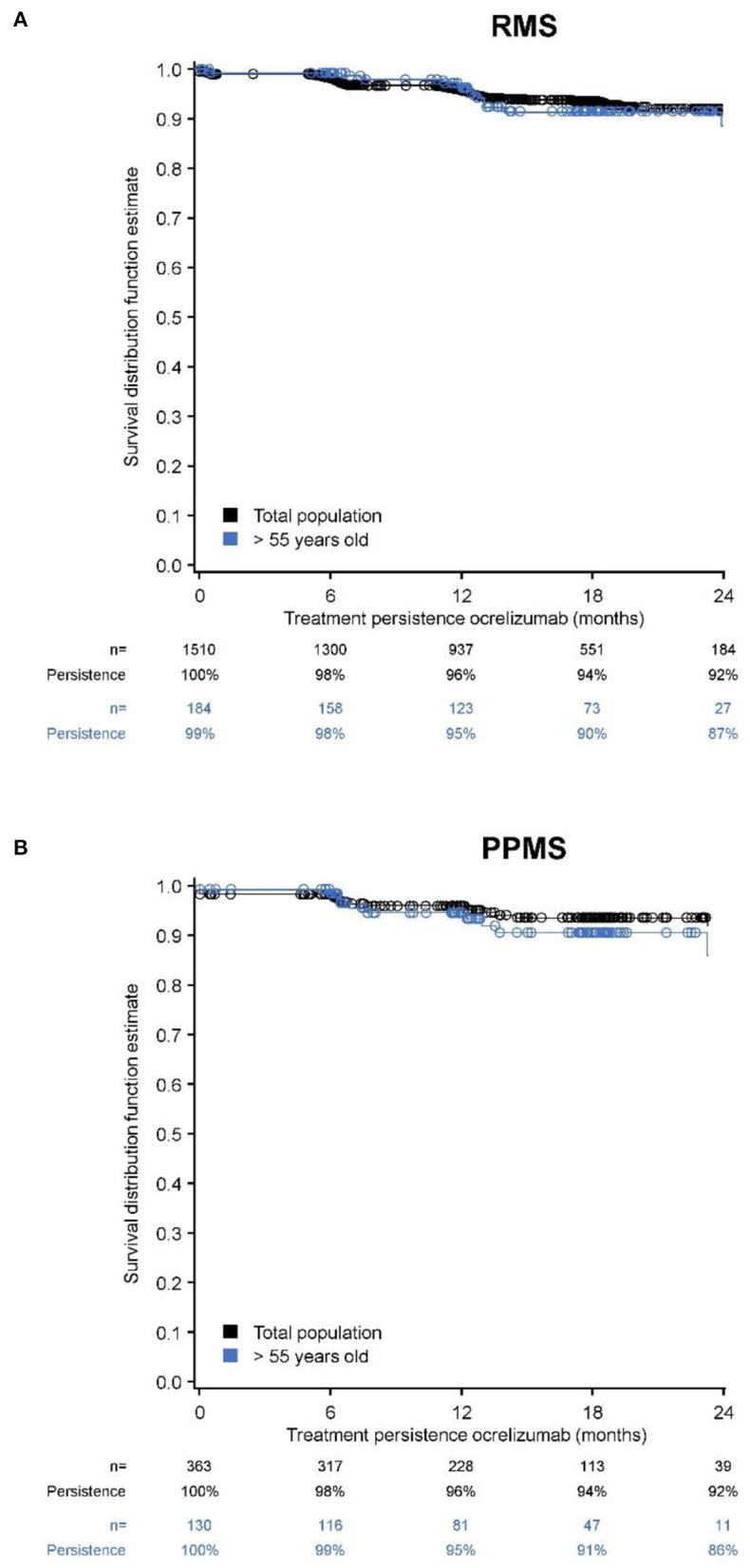
Kaplan-Meier estimates of persistence over 24-months of treatment with ocrelizumab (full analysis set). **(A)** RMS total + >55 years; **(B)** PPMS total + >55 years.

Persistence and adherence were examined in the full analysis set (all patients in the safety set with at least one documentation after start of the therapy; RMS *n* = 1,510; PPMS *n* = 363). Kaplan-Meier analysis showed that patients treated with ocrelizumab achieved 96% and 92% persistence at 12 and 24 months, regardless of MS phenotype (Figures 1A,B). Patients >55 years old (*n* = 184) achieved a 95% and 87% persistence at 12 and 24 months. Patients >55 years with PPMS (*n* = 143) achieved a 95% and 86% persistence at 12 and 24 months.

The median time between infusions was ~6 months, regardless of age group or MS phenotype; and infusion intervals remained stable throughout the treatment duration (Table 4).

**Table 4:** Median time interval between ocrelizumab dosing.

**Dose interval**	**Total RMS (*n =* 1,510)**		**RMS >55 years (*n =* 184)**		**Total PPMS (*n =* 363)**		**PPMS >55 years (*n =* 143)**	
	**n**	**Median, mo (25Q; 75Q)**		**Median, mo (25Q; 75Q)**	**n**	**Median, mo (25Q; 75Q)**		**Median, mo (25Q; 75Q)**
2nd−3rd	1361	5.95 (5.59; 6.18)	163	5.98 (5.70; 6.21)	330	5.95 (5.59; 6.18)	119	5.88 (5.55; 6.18)
3rd−4th	1001	5.98 (5.78; 6.21)	127	5.98 (5.77; 6.21)	246	5.98 (5.75; 6.21)	84	5.98 (5.78; 6.21)
4th−5th	622	5.98 (5.75; 6.21)	86	5.98 (5.75; 6.21	149	5.98 (5.91; 6.21)	53	6.01 (5.98; 6.21)
5th−6th	295	5.98 (5.72; 6.11)	38	5.96 (5.75; 6.01)	67	5.98 (5.75; 6.01)	21	5.88 (5.62; 5.98)

*Mo, months*.

*Data were analyzed in the full analysis set (all enrolled patients who received at least one dose of ocrelizumab with at least one documentation after start of the therapy). Q, quartile*.

## Discussion

Real-world studies provide data to further evaluate the risk/benefit profiles described for new therapies in RCTs. To mitigate potential confounding factors, RCTs include selected patient populations. Real-world studies include diverse patient populations, reflecting features of daily clinical practice. This analysis of the CONFIDENCE study represents a real-world cohort of patients with comorbidities and no limits regarding maximum age or EDSS. According to the later onset of disease, patients with PPMS were on average slightly older and had a higher EDSS than patients with RMS. As ocrelizumab is the only treatment currently available for PPMS, a higher proportion of patients with PPMS were treatment-naïve. Irrespective of MS phenotype, patients >55 years had a higher average baseline EDSS (RMS 4.54; PPMS 4.73) and more comorbidities (RMS 80.5%; PPMS 86.0%) than their respective total phenotypic cohorts.

Although there were no restrictions on the disability status of enrolled patients, average baseline EDSS scores were similar to pivotal trials (4, 5). However, patients in CONFIDENCE were on average older than in pivotal trials, with ~12% of patients with RMS and ~36% of patients with PPMS >55 years (a population excluded from pivotal trials). In addition, patients had longer times since diagnosis, and a greater proportion of patients with RMS had prior MS therapy (~83 vs ~27% in pivotal trials) (4). Moreover, this study includes patients with comorbid conditions who were excluded from RCTs, such as patients with a history of malignancy or congestive heart failure (4, 5). Comorbid conditions often observed in real-world MS populations such as cardiovascular disorders and mood disorders (15) were also seen in CONFIDENCE.

To date, real-world data from large ocrelizumab cohorts that may reflect treatment patterns are rare. CONFIDENCE is a German study and baseline characteristics are largely comparable to another German real-world cohort of ocrelizumab treated patients (16). Compared to a recent smaller US cohort (17), patients with RMS in CONFIDENCE had similar mean EDSS and fewer patients in CONFIDENCE were treated with first-line ocrelizumab. With respect to ocrelizumab treated patients documented in the global MSBase registry (2), CONFIDENCE populations had similar age profiles. Patients with RMS tended to have a higher EDSS and proportions of patients without prior therapy were similar. However, patients with PPMS had a lower EDSS and were more often treatment-naïve.

In alignment with populations reported in large real-world studies examining other highly effective DMTs, the CONFIDENCE population was largely similar regarding age, types of comorbidities, baseline EDSS scores, and the majority of patients had been treated with ≥1 prior MS therapy (3, 18).

No new safety signals were identified in this analysis, where many patients had comorbidities and many RMS patients had multiple previous therapies (~35% had ≥3). Patients with PPMS in CONFIDENCE experienced numerically lower rates of both AEs and SAEs than patients with RMS. Patients with PPMS >55 years experienced SAEs at approximately half the rate (SAE/100 PY) of patients with RMS >55 years. Nevertheless, patients with PPMS comprised a smaller population, and patients with PPMS >55 years were less likely to have previous/multiple previous therapies and had shorter disease durations since diagnosis. As expected, patients >55 years (irrespective of MS phenotype) had higher SAE rates than their respective total cohorts.

Compared with the general population, patients with MS experience infections and hospitalizations due to infections at higher rates (19). Accordingly infections were among the most common AEs reported in CONFIDENCE. However, rates of serious infections remained low. Patients with PPMS had a numerically lower rate of infections and serious infections than patients with RMS. Patients >55 years, however, experienced similar rates of serious infections to that of their overall respective populations. Overall, infections most often reported in CONFIDENCE (respiratory infections and urinary tract infections) were consistent with the described ocrelizumab safety profile (8) and the general MS population (19). The single case of suspected PML in a patient with RMS was considered a carry-over from previous natalizumab treatment.

During this analysis (data cutoff 14/Oct/2020), six patients were reported to have COVID-19 or COVID-19 pneumonia (all with RMS). Two cases were considered serious with only one requiring hospitalization. All patients with known COVID-19 outcomes recovered.

Presented data on COVID-19 are from an early time (pre vaccination era) in the COVID-19 pandemic. A recent analysis (May 2021) using the ocrelizumab post-marketing safety database and clinical trial data show that COVID-19 infections in patients treated with ocrelizumab were mostly mild to moderate, and risk factors known to be associated with severe disease course in the general population were associated with severity in ocrelizumab-treated (20). However, a number of real-world studies (21–24) suggest an increased risk of severe COVID-19 in patients with MS treated with anti-CD20 treatments although subject to potential limitations, including biases, confounding, sample size and data completeness (25). Further analyses are required to understand the risk and severity of COVID-19 in ocrelizumab treated patients.

Another important question will be to determine the clinical protection conferred by SARS-CoV-2 vaccines against severe forms of COVID-19. Attenuated humoral immune response has been associated with ocrelizumab treatment to non-live vaccines (26). However, the development of a protective immune response after vaccination involves a variety of mechanisms, of which T and B cells are variably involved (27). In the context of COVID-19, whether antibody production is the appropriate or sole immune correlate of protection is currently unknown and the role of T or B cell-mediated immunity for effective clinical protection requires additional investigations. Available data report impaired humoral response to SARS-CoV-2 infection or vaccines in ocrelizumab treated patients, but induced robust cellular response (28–35), significantly boosted after a third vaccine dose (36), or preserved against SARS-CoV-2 Delta or Omicron variants (37). Despite the impaired humoral immune response to SARS-CoV2 infection or vaccine, no known correlation with clinical severity has been established, as compensatory cellular-mediated immune response could provide protection against serious complications from COVID-19 infection.

Immunoglobulins and B-cell levels are not routinely checked for in clinical practice and neither the assessment nor the collection of corresponding data are mandatory in this real-world study. Available data collected on immunoglobulins and B-cell levels as part of the CONFIDENCE study are limited in time and potentially biased by lack of systematic data collection across participating centers, therefore they do not allow to assess the association between serious infections, duration of treatment and respective laboratory values.

There was no indication of increased malignancy rates in analyses of the overall ocrelizumab clinical program and post-marketing data compared with matched reference MS and general populations (8, 38). In this analysis of CONFIDENCE, rates of malignancy resembled previously published data from RCTs including open-label extension phases, which were within the expected epidemiological ranges (8, 39).

Persistence and adherence to an effective DMT are associated with lower relapse rates, better clinical outcomes, and reductions in the cost of patient care (40, 41) and can be related to treatment satisfaction and safety (42). Data in CONFIDENCE were consistent with US claims data (43), which show that ocrelizumab has a high persistence. Persistence remained high in patients >55 years across all MS phenotypes. Because there were few discontinuations, no major reasons could be identified. Furthermore, discontinuations due to AEs were rare. Adherence was consistent across MS phenotypes and age groups, with median intervals of ~6 months in between ocrelizumab infusions, in accordance with the regulatory label.

CONFIDENCE is a real-world study and is thus susceptible to the limitations of non-interventional studies (e.g., potential enrollment and channeling biases between cohorts). Efforts to mitigate limitations and biases associated with long-term real-world cohort studies (such as healthy user bias and depletion of susceptibles) included only enrolling patients newly treated with ocrelizumab or selected DMTs. All study sites underwent standardized training and used standardized documentation for the completion of eCRFs at enrollment and for each follow-up assessment, specifically for collecting exposure and outcome variable information. Due to the observational nature of the CONFIDENCE study and spontaneous reporting of AEs, a bias in the reporting of AEs cannot be excluded (e.g., underreporting of non-serious AEs and overrepresentation of SAEs) and information on fatal cases and laboratory values (e.g., Immunoglobulins, B-cell levels) is limited.

Overall, CONFIDENCE represents the use of ocrelizumab in clinical practice and includes patients with physical disability, with comorbid conditions, and patients >55 years. No new safety signals were detected in this analysis, confirming the tolerability and safety of ocrelizumab treatment in a real-world population over a mean of ~1 year of exposure (max 2.5 years). High adherence and persistence to ocrelizumab were observed in patients with RMS or PPMS, and discontinuations were rare due to AE. Further analyses of this large, real-world study will be conducted on a regular basis to provide continuing safety and effectiveness data for the treatment of patients with ocrelizumab for up to 10 years.

## Data Availability Statement

The original contributions presented in the study are included in the article/Supplementary Material, further inquiries can be directed to the corresponding author.

## Ethics Statement

The studies involving human participants were reviewed and approved by Ethikkommission an der Technischen Universität Dresden, Germany (12 February 2018 and 10 April 2019; reference EK 62022018). The patients/participants provided their written informed consent to participate in this study.

## Author Contributions

All authors analyzed and interpreted the data in study, participated in the writing, and approved the final version of this manuscript.

## Funding

The CONFIDENCE study was funded by Roche Pharma AG (Grenzach-Wyhlen, Germany). Financial support for Medical writing services from Ashfield MedComms GmbH (Mannheim, Germany) was provided by Roche Pharma AG (Grenzach-Wyhlen, Germany).

## Conflict of Interest

MW receives research support from the Deutsche Forschungsgemeinschaft (DFG; WE 3547/5-1), from Novartis, TEVA, Biogen-Idec, Roche, Merck and the ProFutura Programm of the Universitätsmedizin Göttingen. MW is serving as an editor for PLoS ONE, received travel funding and/or speaker honoraria from Biogen-Idec, Merck Serono, Novartis, Roche, TEVA, Bayer and Genzyme. MB received honoraria for lecturing, consulting and/or travel expenses for attending meetings from Bayer, Biogen, Boehringer, Bristol Myers Squibb, Coloplast, Daiichi-Sankyo, Das Fortbildungskolleg, Medscape, Merck, Novartis, Roche, Sandoz, Sanofi, and Teva. SM received honoraria for lecturing and travel expenses for attending meetings from Almirall, Amicus Therapeutics Germany, Bayer Health Care, Biogen, Celgene, Diamed, Genzyme, MedDay Pharmaceuticals, Merck Serono, Novartis, Novo Nordisk, ONO Pharma, Roche, Sanofi-Aventis, Chugai Pharma, QuintilesIMS, and Teva, research is funded by the German Ministry for Education and Research (BMBF), Bundesinstitut für Risikobewertung (BfR), Deutsche Forschungsgemeinschaft (DFG), Else Kröner Fresenius Foundation, Gemeinsamer Bundesausschuss (G-BA), German Academic Exchange Service, Hertie Foundation, Interdisciplinary Center for Clinical Studies (IZKF) Muenster, German Foundation Neurology and Alexion, Almirall, Amicus Therapeutics Germany, Biogen, Diamed, Fresenius Medical Care, Genzyme, HERZ Burgdorf, Merck Serono, Novartis, ONO Pharma, Roche and Teva. PD is an employee of F. Hoffmann-La Roche AG and shareholder of F. Hoffmann-La Roche AG. JE is an employee of Roche Pharma AG and shareholder of F. Hoffmann-La Roche AG. SH-S is an employee of Roche Pharma AG. JL is an employee of Roche Pharma AG and shareholder of F. Hoffmann-La Roche AG. EM-LR is an employee of F. Hoffmann-La Roche AG and shareholder of F. Hoffmann-La Roche AG. TZ reports grants and personal fees from Biogen, Roche, TEVA and grants, personal fees from Almirall, Biogen, Celgene, Biogen, Novartis, Merck, TEVA, Janssen, Roche. The authors declare that this study received funding from Roche. The funder had the following involvement in the study: study design, analysis, interpretation of data, the writing of this article and the decision to submit it for publication.

## Publisher's Note

All claims expressed in this article are solely those of the authors and do not necessarily represent those of their affiliated organizations, or those of the publisher, the editors and the reviewers. Any product that may be evaluated in this article, or claim that may be made by its manufacturer, is not guaranteed or endorsed by the publisher.
