# Supplementary material for: Safety, Adherence and Persistence in a Real-World Cohort of German MS Patients Newly Treated With Ocrelizumab: First Insights From the CONFIDENCE Study
Source: Front Neurol. 2022 May 9;13:863105. doi: 10.3389/fneur.2022.863105 (PMC9126090; doi:10.3389/fneur.2022.863105)
Supplement: Supplementary file 1 [file Data_Sheet_1.docx]

**Supplementary information**

**Summaries of selected individual cases**

**Fatal cases and serious adverse events of interest:**

**Fatal Cases**

A 61-year-old male patient died from unknown causes, 20 months after he began ocrelizumab therapy for RMS. Past drugs included interferon beta-1a, mitoxantrone, dimethyl fumarate and methotrexate. Current conditions included trigeminal neuralgia. Concomitant medication included carbamazepine. It is not known if an autopsy was performed.

A 63-year-old male patient died from unknown causes on 19 Dec 2019, 8 months after starting therapy with ocrelizumab for PPMS. The patient was a smoker and had a history of hepatitis B. No further medical or drug history was provided. It is not known if an autopsy was performed.

A 66-year-old male patient committed suicide on 15 Mar 2020, 18 months after starting therapy with ocrelizumab for PPMS. Previous drugs included fampridine, interferon beta-1a and mitoxantrone. No medical history or concomitant medication was reported. He died due to carbon monoxide poisoning.

A 55-year-old male patient died from bronchial carcinoma on 02 Feb 2020. He began therapy with ocrelizumab for RMS on 12 Jun 2018. Past DMT included interferon beta-1b and dimethyl fumarate. Concomitant medication included methylprednisolone, gabapentin, citalopram, enoxaparin and dexamethasone. The patient was a smoker. On 18 Aug 2019 the patient was diagnosed with bronchial carcinoma (grade 4). On 26 Aug 2019 he experienced a generalized seizure and was found to have multiple brain metastases and non-small cell lung cancer. Palliative radiotherapy was commenced. On 04 Oct 2019 the patient was transferred to a hospice where he died on 02 Feb 2020.

A 41-year-old male patient died due to myocarditis on 05 Jan 2020, 9 months after starting therapy with ocrelizumab for RMS. Past DMT included interferon beta-1b, glatiramer acetate, natalizumab and fingolimod. Autopsy showed signs of hypertension and a severe increase in myocardial muscle mass. There were also signs of left sided heart failure, acute brain swelling and lung edema.

**Serious Adverse Events (SAEs):**

*Suspected Progressive Multifocal Leukoencephalopathy (PML)*

A 54-year-old female patient with RMS developed suspected natalizumab carry-over PML after starting therapy with ocrelizumab. The patient was JCV positive when she switched from dimethyl fumarate to natalizumab in May 2015. It was decided to discontinue natalizumab in March 2018 due to an increased JCV antibody titer (last dose administered 05 January 2018). The patient started therapy with ocrelizumab on 28 Mar 2018. He received the second 300 mg dose of ocrelizumab on 12 Apr 2018. On 06 July 2018 MRI was suggestive of PML. The patient did not display clinical symptoms of PML. CSF was negative for JCV DNA (repeated lumbar punctures performed in between July and October 2018). The patient’s clinical course was stable with no new MS lesions evident on MRI, cerebrally or spinally. EDSS decreased from 4.5 on 28 Feb 2018 to 3.5 on 06 July 2018.

*COVID-19*

A 44-year-old female with RMS developed grade 3 COVID pneumonia on 10 March 2020. She was hospitalized and treated with azithromycin, acetylcysteine and hydroxychloroquine. The event resolved and she was discharged from hospital after 11 days. She had no comorbidities. Previous DMTs were interferon beta-1a, dimethyl fumarate and fingolimod. Her last recorded EDSS was 4.0.The patient started treatment with ocrelizumab in August 2018 and received the last infusion prior to the COVID pneumonia in September 2019.Therapy with ocrelizumab therapy was not altered due to this event.

A 52-year-old male with RMS developed grade 2 SARS-COV-2 infection with symptoms of cough and fever on 9 Sept 2020. Due to the mild clinical course of the infection, the patient was not hospitalized. He recovered after 2 weeks. The patient was overweight (BMI 25.6) and had hypercholesterolaemia. Previous DMTs were natalizumab and fingolimod. His baseline EDSS was 6.5.The patient started treatment with ocrelizumab in August 2019 and received the last infusion prior to the onset of the SARS-COV-2-infection in March 2020.Therapy with ocrelizumab was not altered due to this event.

*Encephalitis*

A 61-year-old male with PPMS developed cerebral seizure (grade 2) and was diagnosed with encephalitis (grade 3) approximately one month after the second 300 mg infusion of ocrelizumab. The seizures were successfully managed with lorazepam and levetiracetam. Liquor puncture results were positive for Herpes-simplex-virus 2 and he received acyclovir for 21 days. The patient was discharged following event resolution. Previous drug treatment included mitoxantrone and intrathecal triamcinolone (last dose administered 3 months before starting ocrelizumab). The patient was a smoker and had a history of prostate adenocarcinoma and frontobasal cerebrospinal fluid fistula. Details of the case have been published [1]. Therapy with ocrelizumab was not altered due to this event.

*Herpes Zoster*

A 35-year-old male with RMS developed moderate herpes zoster infection a few weeks after receiving the first 300 mg dose of ocrelizumab. The patient was hospitalized and received treatment with acyclovir. The event resolved and therapy with ocrelizumab was not altered due to the event. Previous DMTs included interferon beta 1a, natalizumab and fingolimod.

*Neuroborreliosis & Meningitis*

A 37-year-old male with RMS developed meningitis and neuroborreliosis. The patient’s medical history included seizures. Medication history included dimethyl fumarate. Prior to starting ocrelizumab, he tested positive for latent tuberculosis infection and started treatment with isoniazid nine days prior to ocrelizumab. Approximately 4 months after the second 300 mg ocrelizumab infusion, the patient developed a severe progressive headache (grade 3). He was admitted to hospital due to significant meningism. Magnetic resonance imaging suggested possible tuberculous meningitis. Neuroborreliosis was diagnosed following lumbar puncture. He received treatment with acyclovir, isoniazid, rifampicin, pyrazinamide and ethambutol and was discharged from the hospital approximately one month later. Treatment with ocrelizumab was not discontinued.

*Endocarditis*

A 52-year-old female with RMS, epilepsy and arrythmia and a history of endocarditis who was taking concurrent torasemide and metoprolol developed recurrent endocarditis one month after the second 300 mg dose of ocrelizumab. Prior DMTs included interferon beta-1a, mitoxantrone and nalatizumab. She was diagnosed with a severe recurrent endocarditis (grade 3 due to Abiotrophia defectiva) on 13 August 2018 and was hospitalized. Transesophageal echocardiography (TEE) showed thickening of the mitral valve. Two days later she was discharged. On 20 November 2018, the patient started treatment with oral clindamycin. On 25 February 2019, the event resolved and clindamycin was discontinued. Therapy with ocrelizumab was continued.

**Supplementary table 1.** All adverse events organized by MedDRA system organ class including the three most-common preferred terms reported during the CONFIDENCE analysis

|  | **Total RMS (n=1702)** | | **RMS >55 years (n=200)** | | **Total PPMS (n=398)** | | **PPMS >55 years (n=143)** | |
| --- | --- | --- | --- | --- | --- | --- | --- | --- |
|  | 1,877 | | 242 | | 452 | | 162 | |
|  | **E*** | **R**** | **E*** | **R**** | **E*** | **R**** | **E*** | **R**** |
| Infections and infestations | 604 | 32.2 | 71 | 29.3 | 89 | 19.7 | 35 | 21.6 |
| Nasopharyngitis | 155 | 8.3 | 12 | 5.0 | 26 | 5.8 | 11 | 6.8 |
| Urinary tract infection | 116 | 6.2 | 20 | 8.3 | 22 | 4.9 | 8 | 4.9 |
| Upper respiratory tract infection | 35 | 1.9 | 4 | 1.7 | 3 | 0.7 | 2 | 1.2 |
| Injury, poisoning and procedural complications | 203 | 10.8 | 31 | 12.8 | 42 | 9.3 | 14 | 8.6 |
| Infusion-related reactions*** | 102 | 5.4 | 13 | 5.4 | 16 | 3.5 | 4 | 2.5 |
| Fall | 30 | 1.6 | 9 | 3.7 | 10 | 2.2 | 3 | 1.9 |
| Contusion | 6 | 0.3 | - | - | 3 | 0.7 | 1 | 0.6 |
| Nervous system disorders | 176 | 9.4 | 30 | 12.4 | 32 | 7.1 | 13 | 8.0 |
| Headache | 45 | 2.4 | 2 | 0.8 | 9 | 2.0 | 3 | 1.9 |
| Dizziness | 19 | 1.0 | 4 | 1.7 | 2 | 0.4 | 2 | 1.2 |
| Multiple sclerosis relapse | 10 | 0.5 | 1 | 0.4 | - | - | - | - |
| Investigations | 165 | 8.8 | 20 | 8.3 | 40 | 8.9 | 14 | 8.6 |
| Alanine aminotransferase increased | 14 | 0.7 | 1 | 0.4 | 1 | 0.2 | - | - |
| Blood immunoglobin M decreased | 13 | 0.7 | - | - | 2 | 0.4 | 1 | 0.6 |
| C-reactive protein increased | 11 | 0.6 | 1 | 0.4 | 1 | 0.2 | - | - |
| General disorders and administration site conditions | 163 | 8.7 | 19 | 7.9 | 34 | 7.5 | 15 | 9.3 |
| Influenza-like illness | 66 | 3.5 | 4 | 1.7 | 9 | 2.0 | 2 | 1.2 |
| Fatigue | 22 | 1.2 | 1 | 0.4 | 5 | 1.1 | 4 | 2.5 |
| Pyrexia | 19 | 1.0 | 1 | 0.4 | 4 | 0.9 | 1 | 0.6 |
| Musculoskeletal and connective tissue disorders | 135 | 7.2 | 18 | 7.4 | 20 | 4.4 | 14 | 8.6 |
| Arthralgia | 20 | 1.1 | 2 | 0.8 | 3 | 0.7 | 2 | 1.2 |
| Pain in extremity | 19 | 1.0 | - | - | 4 | 0.9 | 3 | 1.9 |
| Back pain | 16 | 0.9 | - | - | 4 | 0.9 | 2 | 1.2 |
| Gastrointestinal disorders | 129 | 6.9 | 15 | 6.2 | 15 | 3.3 | 7 | 4.3 |
| Diarrhea | 21 | 1.1 | 2 | 0.8 | 2 | 0.4 | - | - |
| Abdominal pain | 12 | 0.6 | 2 | 0.8 | 2 | 0.4 | - | - |
| Nausea | 12 | 0.6 | - | - | 3 | 0.7 | 2 | 1.2 |
| Skin and subcutaneous tissue disorders | 88 | 4.7 | 3 | 1.2 | 15 | 3.3 | 9 | 5.6 |
| Rash | 15 | 0.8 | 1 | 0.4 | 3 | 0.7 | 2 | 1.2 |
| Alopecia | 13 | 0.7 | 1 | 0.4 | 1 | 0.2 | - | - |
| Eczema | 8 | 0.4 | - | - | - | - | - | - |
| Psychiatric disorders | 70 | 3.7 | 5 | 2.1 | 12 | 2.7 | 7 | 4.3 |
| Depression | 16 | 0.9 | 2 | 0.8 | 4 | 0.9 | 3 | 1.9 |
| Sleep disorder | 12 | 0.6 | 1 | 0.4 | 4 | 0.9 | 2 | 1.2 |
| Middle insomnia | 6 | 0.3 | 1 | 0.4 | - | - | - | - |
| Respiratory, thoracic and mediastinal disorders | 65 | 3.5 | 6 | 2.5 | 17 | 3.8 | 10 | 6.2 |
| Oropharyngeal pain | 20 | 1.1 | 1 | 0.4 | 5 | 1.1 | 4 | 2.5 |
| Cough | 13 | 0.7 | - | - | 3 | 0.7 | 1 | 0.6 |
| Dyspnoea | 6 | 0.3 | 1 | 0.4 | - | - | - | - |
| Blood and lymphatic system disorders | 63 | 3.4 | 7 | 2.9 | 10 | 2.2 | 4 | 2.5 |
| Lymphopenia | 28 | 1.5 | 5 | 2.1 | 2 | 0.4 | 1 | 0.6 |
| Leukopenia**** | 5 | 0.3 | - | - | 1 | 0.2 | 1 | 0.6 |
| Anemia | 4 | 0.2 | 1 | 0.4 | - | - | - | - |
| Metabolism and nutrition disorders | 47 | 2.5 | 5 | 2.1 | 10 | 2.2 | 3 | 1.9 |
| Vitamin D deficiency | 17 | 0.9 | 1 | 0.4 | 7 | 1.5 | 3 | 1.9 |
| Iron deficiency | 10 | 0.5 | 1 | 0.4 | 1 | 0.2 | - | - |
| Decreased appetite | 4 | 0.2 | - | - | 1 | 0.2 | - | - |
| Renal and urinary disorders | 38 | 2.0 | 8 | 3.3 | 10 | 2.2 | 6 | 3.7 |
| Cystitis noninfective | 15 | 0.8 | - | - | 2 | 0.4 | - | - |
| Dysuria | 4 | 0.2 | 1 | 0.4 | - | - | - | - |
| Bladder disorder | 2 | 0.1 | - | - | - | - | - | - |
| Vascular disorders | 33 | 1.8 | 9 | 3.7 | 9 | 2.0 | 4 | 2.5 |
| Hypertension | 12 | 0.6 | 5 | 2.1 | 5 | 1.1 | 2 | 1.2 |
| Deep vein thrombosis | 3 | 0.2 | - | - | - | - | - | - |
| Hot flush | 2 | 0.1 | - | - | 1 | 0.2 | - | - |
| Immune system disorders | 26 | 1.4 | - | - | - | - | - | - |
| Hypogammaglobulinaemia | 5 | 0.3 | - | - | - | - | - | - |
| Seasonal allergy | 5 | 0.3 | - | - | - | - | - | - |
| Anaphylactic reaction***** | 4 | 0.2 | - | - | - | - | - | - |
| Reproductive system and breast disorders | 25 | 1.3 | 2 | 0.8 | 1 | 0.2 | - | - |
| Cervical dysplasia | 3 | 0.2 | - | - | - | - | - | - |
| Menorrhagia | 3 | 0.2 | - | - | - | - | - | - |
| Amenorrhea | 2 | 0.1 | - | - | - | - | - | - |
| Eye disorders | 23 | 1.2 | 3 | 1.2 | 3 | 0.7 | - | - |
| Uveitis | 4 | 0.2 | - | - | - | - | - | - |
| Visual impairment | 3 | 0.2 | - | - | - | - | - | - |
| Eye inflammation | 2 | 0.1 | - | - | - | - | - | - |
| Ear and labyrinth disorders | 22 | 1.2 | 3 | 1.2 | 2 | 0.4 | 2 | 1.2 |
| Vertigo | 6 | 0.3 | 2 | 0.8 | 2 | 0.4 | 2 | 1.2 |
| Middle ear inflammation | 5 | 0.3 | - | - | - | - | - | - |
| Sudden hearing loss | 4 | 0.2 | - | - | - | - | - | - |
| Neoplasms benign, malignant and unspecified (inc cysts and polyps) | 20 | 1.1 | 1 | 0.4 | 5 | 1.1 | 2 | 1.2 |
| Breast cancer female | 2 | 0.1 | - | - | - | - | - | - |
| Leiomyoma | 2 | 0.1 | - | - | - | - | - | - |
| Melanocytic naevus | 2 | 0.1 | - | - | - | - | - | - |
| Pregnancy, puerperium and perinatal conditions | 20 | 1.1 | - | - | - | - | - | - |
| Pregnancy | 16 | 0.9 | - | - | - | - | - | - |
| Abortion spontaneous | 2 | 0.1 | - | - | - | - | - | - |
| Unintended pregnancy | 2 | 0.1 | - | - | - | - | - | - |
| Cardiac disorders | 19 | 1.0 | - | - | 2 | 0.4 | 2 | 1.2 |
| Tachycardia | 4 | 0.2 | - | - | 2 | 0.4 | 2 | 1.2 |
| Bradycardia | 3 | 0.2 | - | - | - | - | - | - |
| Acute myocardial infarction | 2 | 0.1 | - | - | - | - | - | - |
| Not coded | 18 | 1.0 | - | - | 7 | 1.5 | 4 | 2.5 |
| Hepatobiliary disorders | 11 | 0.6 | 3 | 1.2 | 2 | 0.4 | 1 | 0.6 |
| Cholelithiasis | 4 | 0.2 | 1 | 0.4 | 1 | 0.2 | 1 | 0.6 |
| Cholecystitis | 2 | 0.1 | 1 | 0.4 | 1 | 0.2 | - | - |
| Bile duct stone | 1 | <0.1 | - | - | - | - | - | - |
| Endocrine disorders | 7 | 0.4 | 1 | 0.4 | 1 | 0.2 | 1 | 0.6 |
| Hypothyroidism | 4 | 0.2 | - | - | 1 | 0.2 | - | - |
| Hyperthyroidism | 2 | 0.1 | - | - | - | - | 1 | 0.6 |
| Goiter | 1 | <0.1 | 1 | 0.4 | - | - | - | - |
| Surgical and medical procedures | 6 | 0.3 | - | - | 1 | 0.2 | 1 | 0.6 |
| Abortion induced | 4 | 0.2 | - | - | - | - | - | - |
| Abdominal wall operation | 1 | <0.1 | - | - | - | - | - | - |
| Wisdom teeth removal | 1 | <0.1 | - | - | - | - | - | - |
| Congenital, familial and genetic disorders | 5 | 0.3 | 1 | 0.4 | 1 | 0.2 | 1 | 0.6 |
| Dermoid cyst | 1 | <0.1 | - | - | - | - | - | - |
| Factor II deficiency | 1 | <0.1 | - | - | - | - | - | - |
| Factor V Leiden mutation | 1 | <0.1 | - | - | - | - | - | - |

*Total events. **Rate, AEs/100 PY, calculated by dividing total AEs by exposure in 100 PY. ***As of 26 July 2019, infusion-related reactions were only to be recorded if judged as serious or life-threatening. ****Two patients have lymphopenia and one patient has an agranulocytosis reported with the same onset date as the leukopenia. For the remaining patients, leukopenia is reported only. (2 recovered, 1 not recovered and 3 unknown outcomes) *****4 events were reported in 2 patients and all occurred in the context of infusion related reactions. One patient experienced 3 episodes following her first, third and fourth ocrelizumab infusions. In the first two episodes the events resolved on the same day following standard treatment. The third episode resulted in the patient being admitted to hospital where her infusion was administered over 2 days (300mg each day). The event resolved on the day of the second 300mg infusion, the patient was discharged the same day and therapy with ocrelizumab is continuing. The second patient experienced a reaction during the post infusion observation period following her second split dose of ocrelizumab. The patient was treated with intravenous prednisolone and clemastine and did not require withdrawal from the study. Data were analyzed in the safety set, which included all enrolled patients with at least one dose of ocrelizumab; All SOCs shown with most common PT. AEs were classified according to MedDRA versions 23.1. Most common PT according to patients with RMS. E, Total number of events; PPMS, primary progressive multiple sclerosis; PT, preferred term; PY, patient years; R, rate of events by 100 PY RMS, relapsing RM; SOC, system organ class.

**Supplementary table 2**. Infections that occurred during the CONFIDENCE analysis

|  | **Total RMS (n=1702)** | | **RMS >55 years (n=200)** | | **Total PPMS (n=398)** | | **PPMS >55 years (n=143)** | |
| --- | --- | --- | --- | --- | --- | --- | --- | --- |
| Exposure in PY | 1,877 | | 242 | | 452 | | 162 | |
|  | **E*** | **R**** | **E*** | **R**** | **E*** | **R**** | **E*** | **R**** |
| Infections and infestations | 2186 | 116 | 263 | 109 | 380 | 84 | 169 | 104 |
| Nasopharyngitis | 155 | 8.3 | 12 | 5.0 | 26 | 5.8 | 11 | 6.8 |
| Urinary tract infection | 116 | 6.2 | 20 | 8.3 | 22 | 4.9 | 8 | 4.9 |
| Upper respiratory tract infection | 35 | 1.9 | 4 | 1.7 | 3 | 0.7 | 2 | 1.2 |
| Respiratory tract infection | 26 | 1.4 | 3 | 1.2 | 2 | 0.4 | 1 | 0.6 |
| Bronchitis | 23 | 1.2 | 4 | 1.7 | 2 | 0.4 | 2 | 1.2 |
| Sinusitis | 22 | 1.2 | 3 | 1.2 | 2 | 0.4 | - | - |
| Gastrointestinal infection | 20 | 1.1 | - | - | 4 | 0.9 | 2 | 1.2 |
| Oral herpes | 15 | 0.8 | 1 | 0.4 | 2 | 0.4 | - | - |
| Herpes zoster | 12 | 0.6 | 2 | 0.8 | 3 | 0.7 | 2 | 1.2 |
| Pneumonia | 9 | 0.5 | 1 | 0.4 | 2 | 0.4 | - | - |
| Cystitis | 7 | 0.4 | 1 | 0.4 | 1 | 0.2 | - | - |
| Influenza | 7 | 0.4 | 1 | 0.4 | - | - | - | - |
| Pulpitis dental | 7 | 0.4 | 1 | 0.4 | 1 | 0.2 | 1 | 0.6 |
| Viral infection | 7 | 0.4 | 2 | 0.8 | 2 | 0.4 | - | - |
| Herpes simplex | 6 | 0.3 | 3 | 1.2 | - | - | - | - |
| Rhinitis | 6 | 0.3 | 1 | 0.4 | 1 | 0.2 | - | - |
| Vulvovaginal mycotic infection | 6 | 0.3 | - | - | - | - | - | - |
| COVID-19*** | 6 | 0.3 | - | - | - | - | - | - |
| Conjunctivitis | 5 | 0.3 | - | - | 1 | 0.2 | - | - |
| Febrile infection | 5 | 0.3 | 1 | 0.4 | 2 | 0.4 | 1 | 0.6 |
| Infection susceptibility increased | 5 | 0.3 | 1 | 0.4 | 1 | 0.2 | - | - |
| Pyelonephritis | 4 | 0.2 | - | - | - | - | - | - |
| Viral upper respiratory tract infection | 4 | 0.2 | - | - | 2 | 0.4 | - | - |
| Bacteriuria | 3 | 0.2 | - | - | - | - | - | - |
| Herpes virus infection | 3 | 0.2 | - | - | 1 | 0.2 | - | - |
| Otitis media | 3 | 0.2 | - | - | - | - | - | - |
| Appendicitis | 2 | 0.1 | - | - | - | - | - | - |
| Bacterial vaginosis | 2 | 0.1 | - | - | - | - | - | - |
| Erysipelas | 2 | 0.1 | - | - | - | - | - | - |
| Erythema migrans | 2 | 0.1 | - | - | - | - | - | - |
| Fungal infection | 2 | 0.1 | 1 | 0.4 | - | - | - | - |
| Fungal skin infection | 2 | 0.1 | - | - | - | - | - | - |
| Furuncle | 2 | 0.1 | - | - | - | - | - | - |
| Gastroenteritis norovirus | 2 | 0.1 | - | - | - | - | - | - |
| Genital herpes | 2 | 0.1 | 1 | 0.4 | - | - | - | - |
| Hordeolum | 2 | 0.1 | - | - | - | - | - | - |
| Laryngitis | 2 | 0.1 | - | - | - | - | - | - |
| Localized infection | 2 | 0.1 | - | - | - | - | - | - |
| Onychomycosis | 2 | 0.1 | - | - | - | - | - | - |
| Oral candidiasis | 2 | 0.1 | - | - | - | - | - | - |
| Post procedural infection | 2 | 0.1 | - | - | - | - | - | - |
| Tonsillitis | 2 | 0.1 | - | - | - | - | - | - |
| Urosepsis | 2 | 0.1 | 2 | 0.8 | 1 | 0.2 | - | - |
| Vaginal infection | 2 | 0.1 | - | - | - | - | - | - |
| Abscess limb | 1 | <0.1 | 1 | 0.4 | - | - | - | - |
| Acrodermatitis | 1 | <0.1 | - | - | - | - | - | - |
| Acute sinusitis | 1 | <0.1 | - | - | - | - | - | - |
| Bacterial infection | 1 | <0.1 | - | - | - | - | - | - |
| Bacterial vulvovaginitis | 1 | <0.1 | - | - | - | - | - | - |
| Campylobacter infection | 1 | <0.1 | - | - | - | - | - | - |
| Cellulitis | 1 | <0.1 | 1 | 0.4 | - | - | - | - |
| Cholecystitis infective | 1 | <0.1 | - | - | - | - | - | - |
| Diarrhea infectious | 1 | <0.1 | - | - | - | - | - | - |
| Diverticulitis | 1 | <0.1 | - | - | 1 | 0.2 | 1 | 0.6 |
| Endocarditis | 1 | <0.1 | - | - | - | - | - | - |
| Enteritis infectious | 1 | <0.1 | - | - | - | - | - | - |
| Epididymitis | 1 | <0.1 | - | - | - | - | - | - |
| Epstein-Barr virus infection | 1 | <0.1 | - | - | - | - | - | - |
| Eye infection | 1 | <0.1 | - | - | - | - | - | - |
| Eye infection bacterial | 1 | <0.1 | - | - | - | - | - | - |
| Gastroenteritis | 1 | <0.1 | - | - | - | - | - | - |
| Gastroenteritis clostridial | 1 | <0.1 | - | - | - | - | - | - |
| Genital infection | 1 | <0.1 | - | - | - | - | - | - |
| Gingivitis | 1 | <0.1 | - | - | - | - | - | - |
| Helicobacter infection | 1 | <0.1 | - | - | - | - | - | - |
| Infection | 1 | <0.1 | - | - | 1 | 0.2 | 1 | 0.6 |
| Intervertebral discitis | 1 | <0.1 | - | - | - | - | - | - |
| Latent tuberculosis | 1 | <0.1 | - | - | - | - | - | - |
| Lymphangitis | 1 | <0.1 | - | - | - | - | - | - |
| Meningitis | 1 | <0.1 | - | - | - | - | - | - |
| Nasal herpes | 1 | <0.1 | - | - | 1 | 0.2 | 1 | 0.6 |
| Neuroborreliosis | 1 | <0.1 | - | - | - | - | - | - |
| Oral fungal infection | 1 | <0.1 | - | - | - | - | - | - |
| Papilloma viral infection | 1 | <0.1 | - | - | - | - | - | - |
| Paronychia | 1 | <0.1 | - | - | - | - | - | - |
| Periodontitis | 1 | <0.1 | - | - | - | - | - | - |
| Pharyngitis | 1 | <0.1 | - | - | - | - | - | - |
| Pneumonia bacterial | 1 | <0.1 | - | - | - | - | - | - |
| PML | 1 | <0.1 | - | - | - | - | - | - |
| Pustule | 1 | <0.1 | - | - | - | - | - | - |
| Root canal infection | 1 | <0.1 | - | - | - | - | - | - |
| Sepsis | 1 | <0.1 | - | - | - | - | - | - |
| Skin infection | 1 | <0.1 | - | - | - | - | - | - |
| Staphylococcal infection | 1 | <0.1 | - | - | - | - | - | - |
| Subcutaneous abscess | 1 | <0.1 | - | - | 1 | 0.2 |  |  |
| Tinea pedis | 1 | <0.1 | - | - | - | - | - | - |
| Tongue fungal infection | 1 | <0.1 | - | - | - | - | - | - |
| Tooth abscess | 1 | <0.1 | - | - | - | - | - | - |
| Tubo-ovarian abscess | 1 | <0.1 | - | - | - | - | - | - |
| Upper respiratory tract infection bacterial | 1 | <0.1 | - | - | - | - | - | - |
| Ureaplasma infection | 1 | <0.1 | - | - | - | - | - | - |
| Varicella zoster virus infection | 1 | <0.1 | - | - | - | - | - | - |
| Vestibular neuronitis | 1 | <0.1 | 1 | 0.4 | - | - | - | - |
| Viral pharyngitis | 1 | <0.1 | 1 | 0.4 | - | - | - | - |
| Vulvovaginal candidiasis | 1 | <0.1 | 1 | 0.4 | - | - | - | - |
| Borrelia infection | - | - | - | - | 1 | 0.2 | - | - |
| Encephalitis | - | - | - | - | 1 | 0.2 | 1 | 0.6 |
| Wound infection | - | - | - | - | 1 | 0.2 | 1 | 0.6 |

*Total events. **Rate, AEs/100 PY, calculated by dividing total AEs by exposure in 100 PY. ***Includes COVID-19 and COVID-19 pneumonia.

Infections according to MedDRA SOC ‘Infections and infestations’. Data were analyzed in the safety set, which included all enrolled patients with at least one dose of ocrelizumab; AEs were classified according to MedDRA versions 23.1. E, Total number of events; PY, patient years; R, rate of events by 100 PY.

**Supplementary table 3.** MedDRA SOC ‘Neoplasms benign, malignant and unspecified (incl. cysts and polyps)’ during the CONFIDENCE analysis

| **Patients with ≥1 AE; R; events/100 PY** | **Total RMS (n=1702)** | | **RMS >55 years (n=200)** | | **Total PPMS (n=398)** | | **PPMS >55 years (n=143)** | |
| --- | --- | --- | --- | --- | --- | --- | --- | --- |
| Exposure, PY | 1,877 | | 242 | | 452 | | 162 | |
|  | **E*** | **R**** | **E*** | **R**** | **E*** | **R**** | **E*** | **R**** |
| Breast cancer female | 2 | 0.1 | - | - | - | - | - | - |
| Leiomyoma | 2 | 0.1 | - | - | - | - | - | - |
| Melanocytic naevus | 2 | 0.1 | - | - | - | - | - | - |
| Skin papilloma | 2 | 0.1 | - | - | - | - | - | - |
| Adenoma benign | 1 | <0.1 | - | - | - | - | - | - |
| Anogenital warts | 1 | <0.1 | - | - | - | - | - | - |
| Basal cell carcinoma | 1 | <0.1 | - | - | 1 | 0.2 | - | - |
| Bronchial carcinoma | 1 | <0.1 | - | - | - | - | - | - |
| Enchondromatosis | 1 | <0.1 | 1 | 0.4 | - | - | - | - |
| Fibroadenoma of breast | 1 | <0.1 | - | - | - | - | - | - |
| Malignant melanoma | 1 | <0.1 | - | - | 1 | 0.2 | - | - |
| Metastases to the mediastinum* | 1 | <0.1 | - |  | - | - | - | - |
| Metastasis*** | 1 | <0.1 | - | - | - | - | - | - |
| Metastatic malignant melanoma | 1 | <0.1 | - | - | - | - | - | - |
| Thyroid cancer | 1 | <0.1 | - | - | - | - | - | - |
| Uterine leiomyoma | 1 | <0.1 | - | - | - | - | - | - |
| Neoplasm skin | - | - | - | - | 1 | 0.2 | - | - |
| Prostatic adenoma | - | - | - | - | 1 | 0.2 | 1 | 0.6 |
| Squamous cell carcinoma of the skin | - | - | - | - | 1 | 0.2 | 1 | 0.6 |

*Total events. **Rate, AEs/100 PY, calculated by dividing total AEs by exposure in 100 PY. ***In the patient with metastatic malignant melanoma.

Data were analyzed in the safety set, which included all enrolled people with at least one dose of ocrelizumab; AEs were classified according to MedDRA versions 23.1 PY, patient years.

**Supplementary table 4**. Baseline characteristics and risk factors in patients with malignancies during the CONFIDENCE analysis were heterogeneous

| **Malignancy*** | **Phenotype** | **Age (years)** | **EDSS** | **Sex** | **BMI** | **Comorbidities** | **Previous therapies** | **Recorded family history of cancer** | **Risk factors** | **Recovered** | **Cycles** prior to diagnosis** |
| --- | --- | --- | --- | --- | --- | --- | --- | --- | --- | --- | --- |
| **Thyroid cancer** | RMS | 52 | - | F | 32 | - Osteoarthritis - Asthma - Hypothyroidism | - Interferon β1B - Fingolimod - Natalizumab | - | - Obesity | No | 3 |
| **Breast cancer female (invasive mamma carcinoma)** | RMS | 54 | 5.5 | F | - | - | - Glatiramer acetate - Interferon β1A - Natalizumab - Fingolimod | - | - Obesity | No | 2 |
| **Breast cancer female (invasive ductile mamma carcinoma)** | RMS | 53 | 4 | F | 25 | - Paraparesis - Ataxia - Mental disorder due to a general medical condition - Muscle spasticity - Hypertension | - Azathioprine - Glatiramer acetate - Interferon β1B - Interferon β1A IM - Interferon β1A SC - Natalizumab | Father – lung malignant neoplasm  Mother – malignant melanoma | - Tobacco use in the past | No | 2 |
| **Metastatic malignant melanoma***** | RMS | 38 | 6 | F | 23.9 | - Depression | - Interferon β1A IM - Interferon β1A SC - Mitoxantrone - Glatiramer acetate - Natalizumab | - | - None | No | 3 |
| **Bronchial carcinoma** | RMS | 54 | 4.5 | M | - | - Depression - Incontinence | - Interferon β1B - Dimethyl fumerate | - | - Tobacco | Fatal | 2 |
| **Malignant melanoma** | RMS | 45 | 0 | F | 23.8 |  | - Interferon β1B - Natalizumab | - | - None | No | 4 |
| **Basal cell carcinoma** | RMS | 42 | 2 | F | 23.4 | - Irritable bowel syndrome - Vitamin B12 deficiency - Vitamin D deficiency | - Interferon β1A IM - Interferon β1A SC - Natalizumab - Fingolimod | - | - None | Yes | 1 |
| **Squamous cell carcinoma of skin** | PPMS | 60 | 3.5 | F | - | - | - | - | - Occasional alcohol use | No | - |
| **Malignant melanoma** | PPMS | 55 | 4 | F | 27.7 | - Basedow's disease | - | - | - None | Unknown | 3 |
| **Basal cell carcinoma** | PPMS | 44 | 2 | M | 25.0 | - Skin disorder - Vitamin D deficiency | - |  | - Alcohol use | Resolving | 3 |

*Malignant tumors (narrow), patients with ≥1 malignancy. **The first two 300 mg ocrelizumab doses are considered the first cycle. ***Patient also had metastases to the mediastinum.

Data were analyzed in the safety set, which included all enrolled patients with at least one dose of ocrelizumab. F, female; M, male; MS, multiple sclerosis; PPMS, primary progressive MS; RMS, relapsing MS

**Supplementary table 5**. Available information on adverse events (AEs) that led to discontinuation of ocrelizumab in patients with MS

| Patient | AE (preferred term) |
| --- | --- |
| 1 | - Breast cancer female |
| 2 | - Dizziness - Malaise |
| 3 | - Crohn’s disease* |
| 4 | - Expanded disability status scale score increase |
| 5 | - Asthma** |
| 6 | - Tachycardia - Muscle spasms |
| 7 | - Myalgia - Joint Swelling |
| 8 | - Agranulocytosis*** |
| 9 | - Fatigue - Decreased appetite - Pyrexia - Limbic encephalitis - Affective disorder |

*Crohn´s disease was diagnosed in the month after the first infusion with ocrelizumab. **Asthma was reported as a prior and concomitant disease before start of ocrelizumab treatment. ***A 62-year-old female patient taking concomitant mirtazapine developed severe agranulocytosis one year after starting therapy with ocrelizumab. The event resolved one week later. Therapy with ocrelizumab was discontinued 2 months after event resolution. Agranulocytosis is a listed event for mirtazapine. Data were analyzed in the safety set, which included all enrolled patients with at least one dose of ocrelizumab; AEs were classified according to MedDRA versions 23.1 PY, patient years. PY, patient years.

**References**

1. Dudek MIR, Thies K, Kammenhuber S, Bosel J, Rosche J: **HSV-2-encephalitis in a patient with multiple sclerosis treated with ocrelizumab**. *J Neurol* 2019, **266**(9):2322-2323.
